# In Vitro Anti-Inflammatory, Anti-Oxidant, and Cytotoxic Activities of Four *Curcuma* Species and the Isolation of Compounds from *Curcuma aromatica* Rhizome

**DOI:** 10.3390/biom10050799

**Published:** 2020-05-21

**Authors:** Aknarin Pintatum, Wisanu Maneerat, Emilie Logie, Emmy Tuenter, Maria E. Sakavitsi, Luc Pieters, Wim Vanden Berghe, Tawanun Sripisut, Suwanna Deachathai, Surat Laphookhieo

**Affiliations:** 1 Center of Chemical Innovation for Sustainability (CIS) and School of Science, Mae Fah Luang University, Chiang Rai 57100, Thailand; p.aknarin@gmail.com (A.P.); wisanu.man@mfu.ac.th (W.M.); suwanna.dea@mfu.ac.th (S.D.); 2 Medicinal Plants Innovation Center of Mae Fah Luang University, Chiang Rai 57100, Thailand; 3 Lab Protein Chemistry, Proteomics & Epigenetic Signalling (PPES), Department Biomedical Sciences, University of Antwerp, 2610 Wilrijk, Belgium; emilie.logie@uantwerpen.be; 4 Natural Products & Food Research and Analysis (NatuRA), Department of Pharmaceutical Sciences, University of Antwerp, 2610 Wilrijk, Belgium; emmy.tuenter@uantwerpen.be (E.T.); luc.pieters@uantwerpen.be (L.P.); 5 Department of Pharmacognosy and Natural Products Chemistry, Faculty of Pharmacy, National and Kapodistrian University of Athens, Zografou, 15771 Athens, Greece; msakavitsi@pharm.uoa.gr; 6 School of Cosmetic Science, Mae Fah Luang University, Chiang Rai 57100, Thailand; tawanun.sri@mfu.ac.th

**Keywords:** *Curcuma aromatica*, sesquiterpene, anti-inflammatory, luciferase assay, cytotoxicity

## Abstract

The genus *Curcuma* is part of the Zingiberaceae family, and many *Curcuma* species have been used as traditional medicine and cosmetics in Thailand. To find new cosmeceutical ingredients, the in vitro anti-inflammatory, anti-oxidant, and cytotoxic activities of four *Curcuma* species as well as the isolation of compounds from the most active crude extract (*C. aromatica*) were investigated. The crude extract of *C. aromatica* showed 2,2-diphenyl-1-picrylhydrazyl (DPPH) radical scavenging activity with an IC_50_ value of 102.3 μg/mL. The cytotoxicity effect of *C. aeruginosa, C. comosa, C. aromatica*, and *C. longa* extracts assessed with the 3-[4,5-dimethylthiazol-2-yl]-2,5-diphenyl tetrazolium bromide (MTT) assay at 200 μg/mL were 12.1 ± 2.9, 14.4 ± 4.1, 28.6 ± 4.1, and 46.9 ± 8.6, respectively. *C. aeruginosa* and *C. comosa* presented apoptosis cells (57.7 ± 3.1% and 32.6 ± 2.2%, respectively) using the CytoTox-ONE™ assay. Different crude extracts or phytochemicals purified from *C. aromatica* were evaluated for their anti-inflammatory properties. The crude extract of *C. aromatica* showed the highest potential to inhibit NF-κB activity, followed by *C. aeruginosa, C. comosa*, and *C. longa*, respectively. Among the various purified phytochemicals curcumin, germacrone, curdione, zederone, and curcumenol significantly inhibited NF-κB activation in tumor necrosis factor (TNF) stimulated HaCaT keratinocytes. Of all compounds, curcumin was the most potent anti-inflammatory.

## 1. Introduction

The genus *Curcuma* is part of the family Zingiberaceae and over 120 species have been identified [1]. Many *Curcuma* species have been used as traditional medicine for the treatment of various diseases [2], or as ingredients for coloring in cosmetics as well as enhancing food flavors [3,4,5,6]. Previous phytochemical investigations of *Curcuma* species resulted in the isolation and identification of sesquiterpenoids and diarylheptanoids as major constituents and many of them showed promising pharmacological activities including anti-inflammatory activity, cytotoxicity against cancer cell lines, and antioxidant activities [5,6,7,8,9].

*C. aromatica* is widely used in Thai and Chinese traditional medicine for anti-tumor therapy [6], blood stasis [10], throat infections [3], to eliminate body waste, and to promote wound healing [11]. It showed various pharmacological activities such as antioxidant, anti-inflammatory, and anti-carcinogenic activities [12]. The rhizome extract of this plant is well-known as a rich source of sesquiterpenes [5,13]. *C. comosa* has been used in Thai traditional medicine for the alleviation of postpartum uterine pain [14]. This plant showed various biological properties such as antioxidant, anti-inflammatory, insecticidal [15], and inhibitory effects on cell proliferation [16]. Sesquiterpenoids [8] and diarylheptanoids [15] were isolated as major compounds from the rhizome of *C. comosa*. The rhizome of *C. aeruginosa* has been traditionally used for the treatment of asthma, cancer, fever, inflammation, and skin diseases [17]. Pharmacological activities such as antioxidant, anti-inflammatory, and cytotoxic activities have been reported for extracts of this species. [18]. The phytochemical profile of the rhizome of *C. aeruginosa* is characterized by the presence of diarylheptanoids, curcuminoids, and sesquiterpenoids [17,19,20]. *C. longa* is commonly known as turmeric and its rhizome is used as food and in traditional medicine for the treatment of inflammation, infections or tumors, as carminative, and as diuretic [21,22,23]. In this study, we compared in vitro anti-inflammatory and anti-oxidant activity, and cytotoxicity of four *Curcuma* species namely, *C. aromatica, C. comosa, C aeruginosa*, and *C. longa*. In addition, over a dozen compounds were isolated from *C. aromatica* rhizome and its phytochemical profile was compared to that of the other three *Curcuma* species by means of Ultra-Performance Liquid Chromatography–High Resolution Mass Spectrometry (UPLC-HRMS) analysis.

## 2. Materials and Methods

### 2.1. Plant Material

The rhizome of *C. aromatica* (N: 20.1924°, E: 99.4854°), *C. comosa* (N: 20.1922°, E: 99.4852°), and *C. longa* (N: 20.1927°, E: 99.4855°) were collected from Doi Tung, Chiang Rai Province, Thailand in May 2016, while the rhizome of *C. aeruginosa* was purchased from Mae-Ca-Chan local markets, Chiang Rai Province, Thailand in June 2016. Plant authentication was verified by Mr. Martin Van de Bult and voucher specimens (MFU-NPR0192, MFU-NPR0193, MFU-NPR0194, and MFU-NPR0195, respectively) were deposited at the Natural Products Research Laboratory of Mae Fah Luang University.

### 2.2. Chemicals

L-Ascorbic acid, 2,2′ -azino-bis(3-ethylbenzothiazoline-6-sulfonic acid) diammonium salt (ABTS), 2,2-diphenyl-1-picrylhydrazyl (DPPH), 3-[4,5-dimethylthiazol-2-yl]-2,5-diphenyl tetrazolium bromide (MTT), sodium dodecyl sulfate (SDS), and dimethyl sulfoxide (DMSO) were purchased from Sigma-Aldrich (St. Louis, MO, USA). All chemicals and solvents used in this study were of analytical grade.

### 2.3. Extraction

The rhizomes of the four *Curcuma* species were cleaned, chopped, and air-dried at room temperature for three days. The air-dried rhizomes (1 kg) of each plant were macerated in EtOAc (3 × 10 L) at room temperature. The extracts were filtered and evaporated under reduced pressure to obtain the EtOAc extracts of *C. aromatica* (21.67 g), *C. comosa* (24.49 g), *C. aeruginosa* (20.21 g), and *C. longa* (19.76 g). Additionally, dried powder (100 g) of each plant was extracted with 80% ethanol (3 × 500 mL) at room temperature. Removal of the solvent under reduced pressure yielded the crude ethanolic extracts of *C. aromatica* (2.2 g), *C. comosa* (2.5 g), *C. aeruginosa* (2.0 g), and *C. longa* (2.1 g).

### 2.4. Fractionation and Isolation

The EtOAc extract of *C. aromatica* was selected for fractionation and isolation, based on the fact that it showed the most promising biological activities. The EtOAc extract was subjected to quick column chromatography (QCC) over silica gel, eluting with a gradient system of *n*-hexane/EtOAc (100% hexanes to 100% EtOAc) to give 13 fractions (A-M). Fraction B (1.45 g) was further separated by CC over Sephadex LH-20 (100% MeOH) to give compound **1** (4.5 mg). Fraction C (2.26 g) was separated by CC (1:4 CH_2_Cl_2_/*n*-hexane) to give fraction CP21-B5 (443.3 mg), which was further purified by CC over Sephadex LH-20 (100% MeOH) to give compound **7** (15.4 mg). Fraction E (540.1 mg) was separate by CC (1:3 CH_2_Cl_2_/*n*-hexane) to give nine fractions (CP6-01 to CP6-09). Compound **4** (9.9 mg) was obtained from fraction CP6-06 (263.0 mg) by repeated CC over Sephadex LH-20 (1:4 CH_2_Cl_2_/MeOH), while compound **5** (7.0 mg) yielded from fraction CP6-08 (108.5 mg) by repeated CC (1.5:8.5 CH_2_Cl_2_/*n*-hexane). Fraction F (4.05 g) was fractionated by CC (1:19 EtOAc/*n*-hexane) to give fraction CP30-02 (75.1 mg), which was further purified by CC (1:99 acetone/*n*-hexane) to afford compound **6** (5.2 mg). Compound **2** (217.4 mg) was obtained from fraction G (654.7 mg) by CC (2:3 CH_2_Cl_2_/*n*-hexane). Fraction H (3.13 g) was submitted to CC (1:49 EtOAc/*n*-hexane) to give fraction CP32-A (1.12 g), which was further purified by RP-18 (7:3 MeOH/H_2_O) to afford compounds **3** (79.3 mg) and **15** (55.8 mg). Fraction I (957.2 mg) was subjected to CC (1:1 CH_2_Cl_2_/*n*-hexane) to give fraction CP7-2 (198.2 mg), then purified by CC (15:1:34 CH_2_Cl_2_/EtOAc/*n*-hexane) to give compound **8** (9.6 mg). Fraction J (1.30 g) was subjected to CC over Sephadex LH-20 (100% MeOH), followed by CC (3:7 CH_2_Cl_2_/*n*-hexane) to afford compounds **12** (3.1 mg) and **13** (3.1 mg). Fraction K (2.77 g) was fractionated by CC (1:4 EtOAc/*n*-hexane) to give fraction CP35-BC (1.03 g), then repeated CC (1:49 acetone/*n*-hexane and 1:9 CH_2_Cl_2_/*n*-hexane) to afford compound **9** (6.8 mg). Fraction L (2.07 g) was subjected to CC (1:99 acetone/CH_2_Cl_2_) to give compound **11** (31.1 mg) and six fractions (CP17-02 to CP17-07). Compound **14** (31.1 mg) was obtained from fraction CP17-05 (215.7 mg) by CC (1:49 acetone/CH_2_Cl_2_). Compound **10** (5.1 mg) was obtained from fraction CP17-06 (1.56 g) by CC over Sephadex LH-20 (100% MeOH) followed by CC (1:1:3 acetone/EtOAc/*n*-hexane).

### 2.5. Characterization of Curcuma Extracts by UPLC-HRMS

Crude extracts of the four *Curcuma* species, prepared with 80% ethanol/20% water were analyzed by Ultra-Performance Liquid Chromatography–High Resolution Mass Spectrometry (UPLC-HRMS) together with 8 of the 15 purified compounds isolated from *C. aromatica*, in order to determine whether these compounds were present in *C. longa, C. comosa* and *C. aeruginosa* too. Liquid chromatography analysis was performed on an Acquity^®^ UPLC System (Waters, Milford, MA, USA). Detection was carried out on an LTQ-Orbitrap^®^ XL hybrid mass spectrometer equipped with an Electrospray Ionization (ESI) source (Thermo Scientific, Waltham, MA, USA) for accurate mass. Separation was achieved on an Acquity UPLC^®^ Peptide BEH C18 column (2.1 × 100 mm, 1.7 µm, Waters corporation^®^, Wexford, Ireland) using a gradient containing water with 0.1% (*v/v*) formic acid (A) and acetonitrile (B). The gradient elution was performed as follows: 0–2 min eluent B 2%; 2–18 min eluent B 2–100%; 18–20 min eluent B 100%; 21–25 min column equilibration-eluent B 2%. A flow rate of 0.4 mL/min was employed for elution. The column was maintained at 40 °C, the samples at 7 °C, and the flow rate was set to 0.4 mL/min. The 80% ethanol extracts (10 µL at 300 µg/mL) were injected. All samples were analyzed in the full scan *m/z* range of 115–1000, in negative and positive mode at a resolving power of 30,000 and data-dependent MS/MS events were acquired. In both modes the data-dependent acquisition was simultaneously performed using a collision induced dissociation C-trap (CID) with normalized collision energy at 35 V and a mass resolution of 10,000. In negative mode capillary temperature was set to 350 °C and the source voltage was 2.7 kV. Tube lens and capillary voltage were respectively tuned at −100 V and −30 V. In positive mode capillary temperature was set to 350 °C and the source voltage was 3.50 kV. Tube lens and capillary voltage were respectively tuned at +120 V and +40 V. In both modes the arbitrary units were used for sheath gas, auxiliary gas, and sweep gas was nitrogen at (40, 10, 0 arbitrary units, respectively). The control of the system and the spectral interpretation was performed using the XcaliburTM (Version 2.2, Thermo Scientific, Waltham, MA, USA) software.

### 2.6. DPPH Radical-Scavenging Activity Assay

The antioxidant activity was determined by the DPPH radical scavenging assay as described previously, with slight modifications [24]. In brief, 100 μL of extracts and compounds at different concentrations were mixed with 100 μL of 60 μM DPPH methanol solution in a 96-well microplate. The solution was incubated at room temperature in darkness for 30 min, then absorbance was measured at 517 nm. Ascorbic acid was used as positive control. The DPPH radical scavenging activity was expressed as the concentration at 50% inhibition (IC_50_), which was calculated by plotting percent inhibition against concentration of the sample.

### 2.7. ABTS Radical Cation Scavenging Assay

The ABTS radical cation scavenging activity of extracts and compounds was determined using the method described previously [24] with some modifications. The ABTS^∙+^ solution was prepared from the reaction of equal volumes of 7 mM of ABTS and 2.45 mM potassium persulfate in a dark place at room temperature for 16 h before use. Prior to the assay, the ABTS^∙+^ solution was adjusted to the absorbance of 0.70 ± 0.05 at 734 nm with EtOH. Twenty microliters of extracts and compounds at different concentrations were mixed with 180 μL of ABTS^∙+^ solution in a 96-well microplate and incubated at room temperature for 5 min. Next, the absorbance was measured at 734 nm. Ascorbic acid was used as positive control. The ABTS radical cation scavenging activity was expressed as the concentration at 50% inhibition (IC_50_), which was calculated by plotting percent inhibition against concentration of the sample.

### 2.8. Cell Culture

HaCaT keratinocyte cells with a stable transfected NF-κB luciferase reporter gene cassette has previously been described [25]. Cells were cultured in Dulbecco’s modified eagle’s medium, supplemented with 10% fetal bovine serum, 2% of sodium bicarbonate (7.5% solution), 1% of sodium pyruvate (100 mM), and 1% of penicillin–streptomycin (10,000 units/mL). The cells were incubated in a humidified 37 °C, 5% CO_2_ incubator.

### 2.9. MTT Assay

Adverse anti-proliferative or toxic effects of various extracts and purified phytochemicals compounds on HaCaT cells were evaluated by MTT colorimetric assay. Cells were seeded into 96-well plates at 2 × 10^4^ cells/well and incubated under the abovementioned conditions for 24 h. The extracts or pure compounds at different concentrations were added for another 24 h, after which 10 μL of MTT reagent (5 mg/mL) was added to each well and incubated for 4 h. Cells were lysed with 90 µL 10 mM HCl solution containing 10% SDS and OD value was measured at 595 nm with the Envision Plate Reader (Perkin Elmer, USA). Withaferin A was used as positive control.

### 2.10. CytoTox-ONE™ Cytotoxicity Assay

Cell cytotoxicity was measured by determining membrane integrity of HaCaT cells following treatment with crude extracts or purified phytochemicals by means of the CytoTox-ONE™ Assay according to the manufacturer’s instructions (Promega, WI, USA). In brief, cells were plated at 2 × 10^4^ cells/well in 96-well plates and incubated under the above-mentioned conditions for 24 h. Extracts or pure compounds at different concentrations were added to the cells and left to incubate for 24 h at 37 °C and 5% CO_2_. After incubation, the assay plates were transferred to 22 °C for 5 min, 100 μL of the CytoTox-ONE™ reagent was added to all wells and incubated at 22 °C for 10 min. After that, 50 μL of stop solution was added to all wells and plates were shaken at 500 rpm for 10 s. The fluorescence signal was measured with an excitation wavelength of 560 nm and an emission wavelength of 590 nm with the Tecan GENios Microplate Reader (Tecan Trading AG, Männedorf, Switzerland). Withaferin A was used as positive control. The triplicate wells without cells were used as negative control to determine background fluorescence. Vehicle control was triplicate cells with untreated cells, the same solvent used to deliver the test compounds. In addition, 2 μL of lysis solution was used as maximum LDH release control.

### 2.11. Luciferase Assay

NFκB-luciferase-dependent reporter assays were performed in HaCaT cells stably expressing p(NFκB)_3_50-luc as previously described [25]. In brief, cells were plated at a density of 10^5^ cells/well in 24-well plates and grown overnight. Cells were subsequently treated with a dose range of crude extracts or purified compounds for 2 h, followed by TNF stimulation (2 ng/mL) for 6 h. Finally, cells were lysed in 1 X lysis buffer (25 mM Tris-phosphate (pH 7.8), 2 mM DTT, 2 mM CDTA, 10% glycerol, and 1% Triton X-100) and 25 µL of lysate was assayed for luciferase activity by adding 50 µL of luciferase substrate (1 mM luciferin or luciferin salt, 3 mM ATP, and 15 mM MgSO_4_ in 30 mM HEPES buffer, pH 7.8). After 10 s of mixing, bioluminescence was measured for 1 s using the Envision multilabel reader (Perkin Elmer, Waltham, MA, USA). Withaferin A was used as positive control.

### 2.12. Data Analysis

All analyses were performed in triplicate and data were expressed as means ± standard deviation (SD) from at least three independent biological experiments. The results were analyzed by one-way analysis of variance (ANOVA) with the Dunnett test, significant difference (*p* < 0.05) using IBM SPSS Statistics, version 23 (IBM Crop.).

## 3. Results and Discussion

### 3.1. Isolation of Compounds

**Figure 1:**
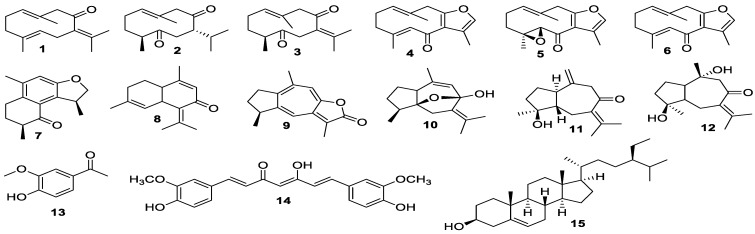
Structures of compounds isolated from *C. aromatica* rhizome.

The EtOAc extract of *C. aromatica* was fractionated by column chromatography to afford 15 known compounds (Figure 1). The compounds were identified as germacrone (**1**) [25], curdione (**2**) [26], dehydrocurdione (**3**) [25], furanodienone (**4**) [27], zederone (**5**) [28], curzerenone (**6**) [27], curzeone (**7**) [29], comosone II (**8**) [30], gweicurculactone (**9**) [31], curcumenol (**10**) [25], isoprocurcumenol (**11**) [32], zedoarondiol (**12**) [33], vanillin (**13**) [34], curcumin (**14**) [35], and *β*-sitosterol (**15**) [36] by comparison of their spectroscopic data with those reported in the literature. Sesquiterpenes **7** and **8** were isolated from the rhizome of *C. aromatica* for the first time, while all remaining sesquiterpenes were similar to previous reports [5,13].

### 3.2. Characterization of Curcuma Extracts by UPLC-HRMS

Eight of the purified compounds, germacrone (**1**), curdione (**2**), dehydrocurdione (**3**), zederone (**5**) curcumenol (**6**), zedoarondiol (**12**), curcumin (**14**), and *β*-sitosterol (**15**), were analyzed by UPLC-HRMS, together with the 80% EtOH extracts of *C. aromatica*, *C. longa*, *C. comosa*, and *C aeruginosa* (Figure S1, Supplementary Material). Except for compounds **12** and **15**, all compounds were detected in ESI^+^ mode, while **5** and **13** could be detected in ESI^+^ and ESI^−^ mode. Table 1 shows the retention time and MS data obtained for the purified compounds. In addition, it is indicated whether these compounds could be detected in the crude extracts. Compounds **12** and **15** were not clearly detected in either of the detection modes, possibly due to poor ionization properties or their low abundance.

As expected, all six detected compounds were found in the crude extract of C. aromatica, since the compounds were purified from this Curcuma species as described in Section 2.1 and Section 3.1 Also C. longa was found to contain these six compounds. Five out of six compounds could be identified in the 80% EtOH extracts of C. aeruginosa; only curcumin (**13**) was found to be absent in this Curcuma species. The C. comosa extract did not contain curcumin either, nor did it contain curcumenol. Our results about the phytochemical composition of different Curcuma species are in line with results reported by other groups [26,27].

### 3.3. Antioxidant Activity

**Figure 2:**
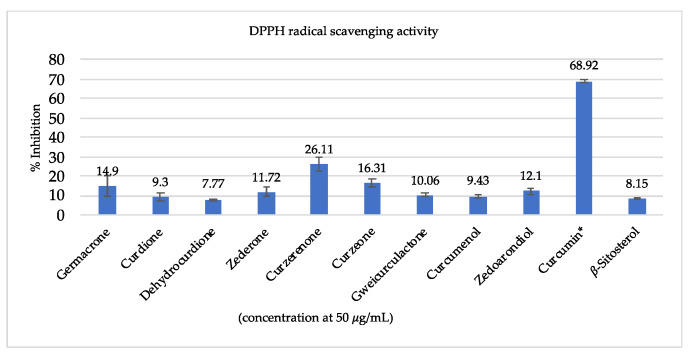
2,2-Diphenyl-1-picrylhydrazyl (DPPH) radical scavenging activity of compounds isolated from *C. aromatica*, * = concentration of 25 μg/mL.

The antioxidant radical scavenging activity of extracts were evaluated using DPPH and ABTS assays (Table 2), and purified compounds were tested in the DPPH assay as shown in Figure 2. Regarding antioxidant activity, the *C. aromatica* extract showed the most promising IC_50_ values (102.4 ± 1.9, 127.0 ± 1.9 μg/mL), followed by *C. longa* (134.9 ± 1.5, 170.8 ± 1.6 μg/mL), *C. comosa* (137.7 ± 5.2, 171.9 ± 1.9 μg/mL), and *C. aeruginosa* (187.4 ± 22.1, 217.9 ± 1.8 μg/mL). Ascorbic acid was used as positive control, with IC_50_ values of 1.80 ± 0.01 and 5.2 ± 0.8 for DPPH and ABTS assay, respectively. In addition, curcumin exhibited strong antioxidant activity with 68.9% ± 0.6% percent inhibition of at 25 μg/mL, whereas other compounds showed moderate activities, see Figure 2. Since curcumin was only detected in *C. aromatica* and *C. longa* and not in *C. comosa* and *C. aeruginosa*, the activity of the first two extracts may in part be attributed to the presence of curcumin. However, since *C. comosa* showed antioxidant activity similar to *C. longa*, and *C. aeruginosa* showed significant antioxidant activity too, curcumin cannot be the only active compound and other constituents might also contribute too to overall antioxidant activity.

### 3.4. Cell Viability and Cytotoxicity

**Figure 3:**
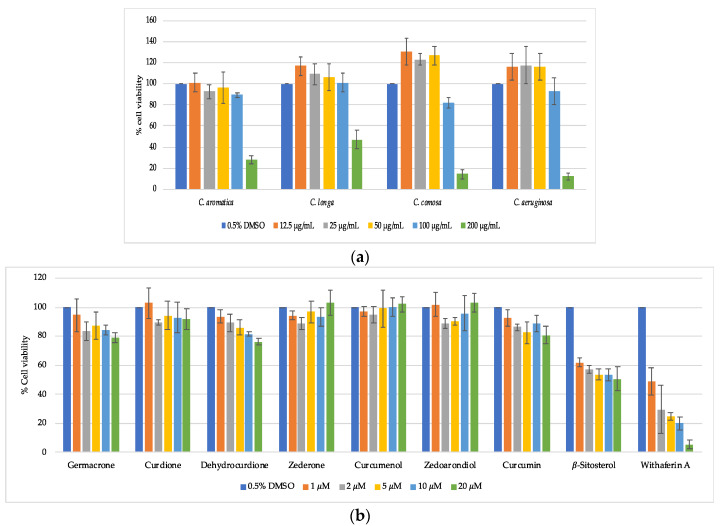
(**a**) Relative HaCaT viability by increasing concentrations of four *Curcuma* species. (**b**) Relative HaCaT viability (%) by increasing concentrations of pure compounds isolated from *C. aromatica* and the reference cytotoxic anti-cancer compound withaferin A in HaCaT cells.

**Figure 4:**
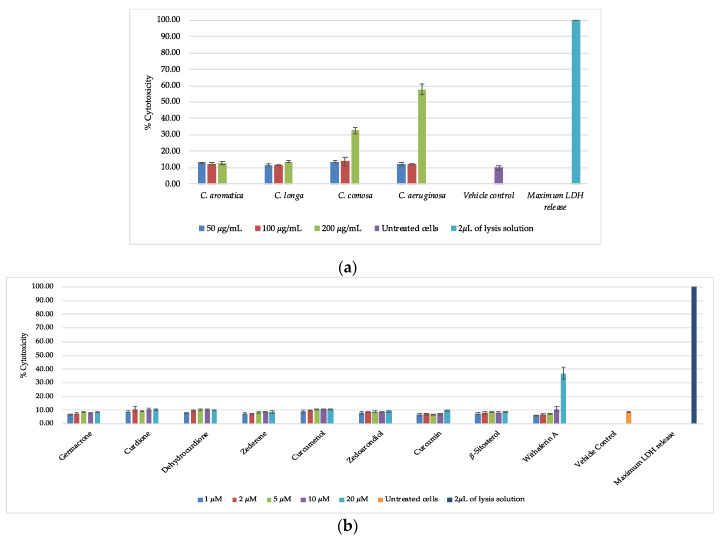
Disruption of membrane integrity measured by the release of lactate dehydrogenase (LDH) (CytoTox-ONE™). (**a**) Relative cytotoxicity (%) of four *Curcuma* species in HaCaT cells. (**b**) Relative cytotoxicity (%) of pure compounds isolated from *C. aromatica* and the reference cytotoxic anti-cancer compound withaferin A in HaCaT cells.

Cell viability and cytotoxicity of crude extracts and pure compounds were assessed by MTT assay and the CytoTox-ONE™ Homogeneous Membrane Integrity Assay using HaCaT keratinocyte cells, respectively. The MTT colorimetric assay estimates the number of viable cells based on the ability of mitochondrial enzymes to reduce the tetrazolium dye MTT to a purple colored formazan [37], whereas the CytoTox-ONE™ assay is a fluorometric-based method to detect loss of membrane integrity of dying cells. MTT results showed that exposure to 200 μg/mL of *C. aeruginosa, C. comosa, C. aromatica*, or *C. longa* extract inhibited the growth of cells, with relative percentages of cell viability being 12.1 ± 2.9, 14.4 ± 4.1, 28.6 ± 4.1, and 46.9 ± 8.6, respectively (Figure 3a). Interestingly, CytoTox-ONE™ showed a slightly different outcome with estimated cell death being lower compared to the MTT results. Treatment of HaCaT cells with 200 µg/mL concentrations of *C. aeruginosa* and *C. comosa* extract resulted in 57.7 ± 3.1% and 32.6 ± 2.2% cell death respectively, while no cytotoxicity could be observed with *C. aromatica* and *C. longa* treatments at the same concentration (Figure 4a). This suggests that all extracts mainly affect mitochondrial reduction capacity and cell proliferation, and that only *C. aeruginosa* and *C. comosa* extracts negatively impact membrane integrity at concentrations above 100 μg/mL [38,39,40]. In contrast, none of the purified phytochemicals inhibit cell viability (MTT) or cytotoxicity (CytoTox-One™) at concentrations 1–20 μM, whereas a reference cytotoxic anti-cancer compound withaferin A [28] dose dependently kills the HaCaT cells, as shown in Figure 3b and Figure 4b.

### 3.5. Anti-Inflammatory Activity

**Figure 5:**
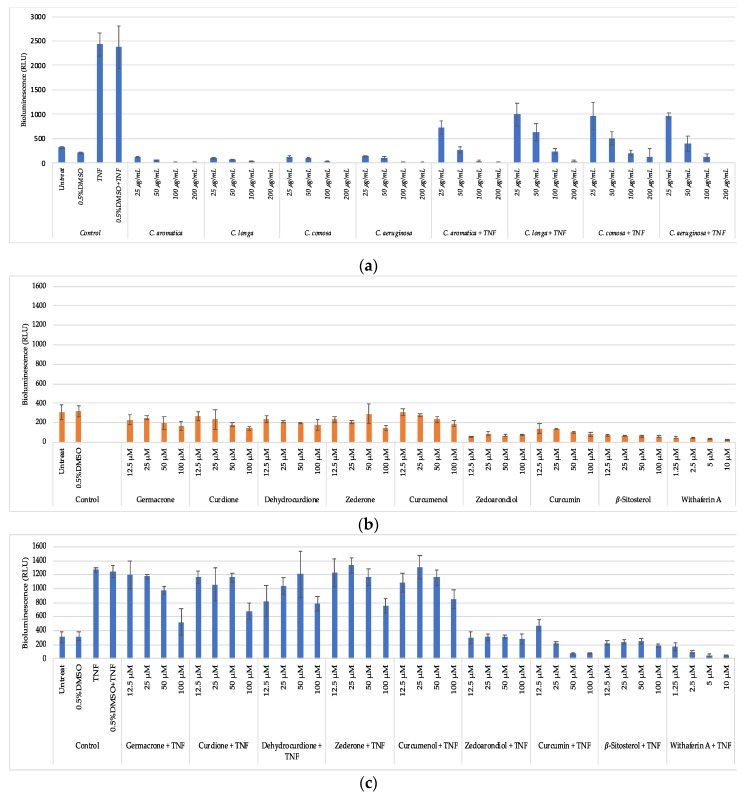
Anti-inflammatory effects of four *Curcuma* species and pure compounds isolated from *C. aromatica* measured in HaCaT NF-κB reporter gene cells. (**a**) Dose dependent effects of crude extracts of *Curcuma* species on basal and inflammation induced NF-κB reporter gene (luciferase relative light units) expression. (**b**) Dose dependent effect of pure compounds isolated from *C. aromatica* and the reference NF-κB inhibitor compound (withaferin A) on basal and inflammation induced NF-κB reporter gene (luciferase relative light units) expression. (**c**) Dose dependent effect of pure compounds isolated from *C. aromatica* and the reference NF-κB inhibitor compound (withaferin A) on basal and inflammation induced NF-κB reporter gene (luciferase relative light units) expression.

HaCaT NF-κB reporter gene cells were left untreated or pretreated for 2 h with various crude extracts or its purified phytochemicals, followed by 3 h combination treatment with the pro-inflammatory stimulus TNF. After 5 h treatment, cells were lysed and corresponding luciferase reporter gene activity was measured in lysates in presence of ATP/luciferin reagent (Promega, WI, USA) by measuring the total emitted bioluminescence (relative light units, RLU) during 30s (Envision multiplate reader, Perkin Elmer). As expected, and as shown in Figure 5a, the proinflammatory NF-κB activator TNF strongly increases luciferase gene expression in HaCaT NF-κB reporter cells, as compared to the control samples without TNF. Upon combination treatment of the different extracts with TNF, we observed dose dependent decrease of luciferase gene expression for all four extracts, suggesting anti-inflammatory effects on NF-κB activity. *C. aromatica* showed the strongest anti-inflammatory NF-κB effects, followed by *C. aeruginosa, C. comosa*, and *C. longa*, respectively. 

Next, stable phytochemicals isolated in sufficient quantities isolated from *C. aromatica* were further evaluated for their NF-κB inhibiting activity in TNF stimulated HaCaT keratinocytes, as compared to the reference inhibitor compound withaferin A [41]. As shown in Figure 5b, curcumin was found to be the most potent NF-κB inhibitor, although less potent the reference NF-κB inhibitor withaferin A, in line with previous research [11,41]. *C. aromatica*, which contains curcumin, indeed was the most potent NF-kB inhibiting extract. Thus, it’s traditional use in the prevention and treatment of inflammatory diseases may be justified. However, the other three extracts, of which *C. longa* contains curcumin, whereas *C. comosa* and *C. aeruginosa* do not, show a comparable activity. This suggests that besides curcumin, additional constituents may be responsible for NF-κB inhibition in *C. comosa* and *C. aeruginosa* extracts. Indeed, germacrone, curdione, zederone, and curcumenol show moderate inhibition of NF-κB reporter gene expression in TNF stimulated HaCaT keratinocytes too. In addition, zedoarondiol and *β*-sitosterol show strong NF-κB inhibition, although they may be low abundant, since UPLC-HRMS analysis failed to detect significant amounts in the four extracts.

## 4. Conclusions

Sesquiterpenes are major bioactive constituents in the rhizome extract of *C. aromatica*. Of the four *Curcuma* species, *C. aromatica*, with its secondary metabolite curcumin, showed the highest antioxidant activity and most potent anti-inflammatory properties with the lowest toxicity. Besides curcumin, we purified additional anti-inflammatory bioactives in *C. aromatica, C. aeruginosa, C. comosa*, and *C. longa*, such as germacrone, curdione, zederone, curcumenol, zedoarondiol, and *β*-sitosterol present, which deserve further investigation.

In conclusion, our results suggest that the rhizome of *C. aromatica* holds promise to be developed as a safe cosmeceutical or functional skin care products for anti-aging and to reduce inflammatory skin irritation.

## Figures and Tables

**Table 1:** Chromatographic and spectral data, obtained with Ultra-Performance Liquid Chromatography–High Resolution Mass Spectrometry (UPLC-HRMS)analysis.

			ESI^+^					ESI^−^					Present in Extract			
Compound	Mol. Formula	RT (min)	Measured *m/z*	Ion	Calculated *m/z*	Δ (ppm)	MS fragments	Measured *m/z*	Ion	Calculated *m/z*	Δ (ppm)	MS fragments	*C. aromatica*	*C. aeruginosa*	*C. comosa*	*C. longa*
Germacrone (**1**)	C_15_H_22_O	13.8	219.1751	[M + H]^+^	219.1749	0.91		n.d.					x	x	x	x
Curdione (**2**)	C_15_H_24_O_2_	11.8	237.1858	[M + H]^+^	237.1855	1.26		n.d.					x	x	x	x
Dehydrocurdione (**3**)	C_15_H_22_O_2_	10.9	235.1703	[M + H]^+^	235.1698	2.13		n.d.					x	x	x	x
Zederone (**5**)	C_15_H_18_O_3_	11.2	247.1339	[M + H]^+^	247.1334	2.02		245.1180	[M − H]^−^	245.1178	0.82		x	x	x	x
Curcumenol (**6**)	C_15_H_22_O_2_	11.1	235.1701	[M + H]^+^	235.1698	1.28	217.1593; 199.1486; 189.1642; 177.1277	n.d.					x	x		x
Curcumin (**13**)	C_21_H_20_O_6_	11.1	369.1345	[M + H]^+^	369.1338	1.90	285.1129; 245.1814; 175.0756	367.1181	[M − H]^−^	367.1182	−0.27	217.0504, 173.0608	x			x

**Table 2:** Antioxidant activities of EtOH extract from the rhizome of *C. aromatica, C. longa, C. comosa*, and *C. aeruginosa*.

Sample	Antioxidant (IC_50_, μg/mL)	
	DPPH	ABTS
*C. aromatica*	102.4 ± 1.9	127.0 ± 1.9
*C. longa*	134.9 ± 1.5	170.8 ± 1.6
*C. comosa*	137.7 ± 5.2	171.9 ± 1.9
*C. aeruginosa*	187.4 ± 22.1	217.9 ± 1.8
Ascorbic acid	1.80 ± 0.01	5.2 ± 0.8

Note: Values are the mean ± SD, n = 3; DPPH: 2,2-diphenyl-1-picrylhydrazyl; ABTS: 2,2′-azino-bis(3-ethylbenzothiazoline-6-sulfonic acid) diammonium salt.

## Supplementary Materials

The following are available online at https://www.mdpi.com/2218-273X/10/5/799/s1, Figure S1: UPLC-HRMS chromatograms of four Curcuma extracts.

## References

[1] Mishra S., Verma S.S., Rai V., Awasthee N., Arya J.S., Maiti K.K., Gupta S.S. (2019). Curcuma raktakanda induces apoptosis and suppresses migration in cancer cells: Role of reactive oxygen species. Biomolecules.

[2] Srivilai J., Rabgay K., Knorana N., Waranuch N., Nuengchamnong N., Wisuitiprot W., Chuprajob T., Changtam C., Suksamrarn A., Chavasiri W. (2017). Anti-androgenic curcumin analogues as steroid 5-alpha reductase inhibitors. Med. Chem. Res..

[3] Dong S., Luo X., Liu Y., Zhang M., Li B., Dai W. (2018). Diarylheptanoids from the root of *Curcuma aromatica* and their antioxidative effects. Phytochem. Lett..

[4] Chan E.W.C., Lim Y.Y., Wong L.F., Lianto F.S., Wong S.K., Lim K.K., Joe C.E., Lim T.Y. (2008). Antioxidant and tyrosinase inhibition properties of leaves and rhizomes of ginger species. Food Chem..

[5] Bamba Y., Yun Y.S., Kunugi A., Inoue H. (2011). Compounds isolated from *Curcuma aromatica* Salisb. inhibit human P450 enzymes. J. Nat. Med..

[6] Liu B., Gao Y.Q., Wang X.M., Wang Y.C., Fu L.Q. (2014). Germacrone inhibits the proliferation of glioma cells by promoting apoptosis and inducing cell cycle arrest. Mol. Med. Rep..

[7] Keeratinijakal V., Kongkiatpaiboon S. (2017). Distribution of phytoestrogenic diarylheptanoids and sesquiterpenoids components in *Curcuma comosa* rhizomes and its related species. Rev. Bras. Farm..

[8] Qu Y., Xu F., Nakamura S., Matsuda H., Pongpiriyadacha Y., Wu L., Yoshikawa M. (2009). Sesquiterpenes from *Curcuma comosa*. J. Nat. Med..

[9] Suksamrarn A., Ponglikitmongkol M., Wongkrajang K., Chindaduang A., Kittidanairak S., Jankam A., Yingyongnarongkul B., Kittipanumat N., Chokchaisiri R., Khetkam P. (2008). Diarylheptanoids, new phytoestrogens from the rhizomes of *Curcuma comosa*: Isolation, chemical modification and estrogenic evaluation. Bioorg. Med. Chem..

[10] Dong S., Li B., Dai W., Wang D., Qin Y., Zhang M. (2017). Sesqui- and diterpenoids from the radix of *Curcuma aromatica*. J. Nat. Prod..

[11] Ramsewak R.S., Dewitt D.L., Nair M.G. (2000). Cytotoxicity, antioxidant and anti-inflammatory activities of Curcumins I-III from *Curcuma longa*. J. Phytomed..

[12] Booker A., Frommenwiler D., Johnston D., Umealajekwu C., Reich E., Heinrich M. (2014). Chemical variability along the value chains of turmeric (*Curcuma longa*): A comparison of nuclear magnetic resonance spectroscopy and high performance thin layer chromatography. J. Ethnopharmacol..

[13] Agnihotri V.K., Thakur S., Pathania V., Chand G. (2014). A new dihomosesquiterpene, termioic acid A, from *Curcuma aromatica*. Chem. Nat. Compd..

[14] Keeratinijakal V., Kladmook M., Laosatit K. (2010). Identification and characterization of *Curcuma comosa* Roxb., phytoestrogens-producing plant, using AFLP markers and morphological characteristics. J. Med. Plants Res..

[15] Jurgens T.M., Frazier E.G., Schaeffer J.M., Jones T.E., Zink D.L., Borris R.P. (1994). Novel nematocidal agents from *Curcuma comosa*. J. Nat. Prod..

[16] Jariyawat S., Thammapratip T., Suksen K., Wanitchakool P., Nateewattana J., Chairoungdua A., Suksamrarn A., Piyachaturawat P. (2011). Induction of apoptosis in murine leukemia by diarylheptanoids from *Curcuma comosa* Roxb. Cell Biol. Toxicol..

[17] Simoh S., Zainal A. (2015). Chemical profiling of *Curcuma aeruginosa* Roxb. rhizome using different techniques of solvent extraction. Asian Pac. J. Trop. Biomed..

[18] Waras N., Nurul K., Muhamad S., Maria B., Ardyani I.D.A.A.C. (2015). Phytochemical screening, antioxidant and cytotoxic activities in extracts of different rhizome parts from *Curcuma aeruginosa* Roxb. Int. J. Res. Ayurveda Pharm..

[19] Jose S., Thamas T.D. (2014). Comparative phytochemical and anti-bacterial studies of two indigenous medicinal plants *Curcuma caesia* Roxb. and *Curcuma aeruginosa* Roxb. Int. J. Green Pharm..

[20] Takano I., Yasuda I., Takeya K., Itokawa H. (1995). Guaiane sesquiterpene lactones from *Curcuma aeruginosa*. Phytochemistry.

[21] Li S., Yuan W., Deng G., Wang P., Yang P., Aggarwal B.B. (2011). Chemical composition and product quality control of turmeric (*Curcuma longa* L.). Pharm. Crop..

[22] Priya R., Prathapha A., Raghu K.G., Menon A.N. (2012). Chemical composition and in vitro antioxidative potential of essential oil isolated from *Curcuma longa* L. leaves. Asian Pac. J. Trop. Biomed..

[23] Gounder D.K., Lingamallu J. (2012). Comparison of chemical composition and antioxidant potential of volatile oil from fresh, fried and cured turmeric (*Curcuma longa*) rhizomes. Ind. Crop. Prod..

[24] Kanlayavattanakul M., Lourith N. (2011). Sapodilla seed coat as a multifunctional ingredient for cosmetic applications. Process. Biochem..

[25] Firman K., Kinoshita T., Itai A., Sankawa U. (1988). Terpenoids from *Curcuma heyneana*. Phytochemistry.

[26] Harimaya K., Gao J.F., Ohkura T., Kawamata T., Iitaka Y., Guo Y.T., Inayama S. (1991). A series of sesquiterpenes with a 7α-isopropyl side chain and related compounds isolated from *Curcuma wenyujin*. Chem. Pharm. Bull..

[27] Hikino H., Konno C., Agatsuma K., Takemoto T., Horibe I., Tori K., Ueyama M., Takeda K. (1975). Sesquiterpenoids part XLVII structure configuration conformation and thermal rearrangement of furanodienone, isofuranodienone, curzerenone, epicuraerenone, and pyrocurzerenone, sesquiterpenoids of *Curcuma zedoaria*. J. Chem. Soc..

[28] Shibuya H., Hamamoto Y., Cai Y., Kitagawa I. (1987). A reinvestigation of the structure of zederone, a furanogermacrane-type sesquiterpene from zedoary. Chem. Pharm. Bull..

[29] Shiobara Y., Asakawa Y., Kodama M., Takemoto T. (1986). Zedoarol, 13-hydroxygermacrone and curzeone, three sesquiterpenoids from *Curcuma zedoaria*. Phytochemistry.

[30] Xu F., Nakamura S., Qu Y., Matsuda H., Pongpiriyadacha Y., Wu L., Yoshikawa M. (2008). Structures of new sesquiterpenes form *Curcuma comosa*. Chem. Pharm. Bull..

[31] Hamdi O.A.A., Ye L.J., Kamarudin M.N.A., Hazni H., Paydar M., Looi C.Y., Shilpi J.A., Kadir H.A., Awang K. (2015). Neuroprotective and antioxidant constituents from *Curcuma zedoaria* rhizomes. Rec. Nat. Prod..

[32] Kuroyanagi M., Ueno A., Koyama K., Natori S. (1990). Structures of sesquiterpenes of *Curcuma aromatica* Salisb. II. Studies on minor sesquiterpenes. Chem. Pharm. Bull..

[33] Kuroyanagi M., Ueno A., Ujiie K., Sato S. (1987). Structures of sesquiterpenes from *Curcuma aromatica* Salisb. Chem. Pharm. Bull..

[34] Bandyopadhyay B., Banik B.K. (2012). Bismuth nitrate-induced microwave-assisted expeditious synthesis of vanillin from curcumin. Org. Med. Chem. Lett..

[35] Payton F., Sandusky P., Alworth W.L. (2007). NMR study of the solution structure of curcumin. J. Nat. Prod..

[36] Pierre L.L., Moses M.N. (2015). Isolation and characterization of stigmasterol and β-sitosterol from *Odontonema strictum* (Acanthaceae). J. Innov. Pharm. Biol. Sci..

[37] Chirumamilla C.S., Palagani A., Kamaraj B., Declerck K., Verbeek M.W.C., Oksana R., Bosscher K.D., Bougarne N., Ruttens B., Gevaert K. (2017). Selective glucocorticoid receptor properties of GSK886 analogs with cysteine reactive warheads. Front. Immunol..

[38] Sun W., Wang S., Zhao W., Wu C., Guo S., Hongwei G., Hongxun T., Lu J.J., Wang Y., Chen X. (2016). Chemical constituents and biological research on plants in the genus *Curcuma*. Food Sci. Nutr..

[39] Kannamangalam U., Varakumar S., Singhal R.S. (2019). A comparative account of extraction of oleoresin from *Curcuma aromatica* Salisb by solvent and supercritical carbon dioxide: Characterization and bioactivities. J. Food Sci. Technol..

[40] Hassannia B., Logie E., Vandenabeele P., Berghe T.V. (2020). Withaferin A: From ayurvedic folk medicine to preclinical anti-cancer drug. Biochem. Pharmacol..

[41] Kaileh M., Berghe W.V., Heyerick A., Horion J., Piette J., Libert C., Keukeleire D.D., Essawi T., Haegeman G. (2006). Withaferin A strongly elicits lkB kinase *β* hyperphosphorylation concomitant with potent inhibition of its kinase activity. J. Biol. Chem..

