# Supplementary figures and images for: In Vitro Anti-Inflammatory, Anti-Oxidant, and Cytotoxic Activities of Four Curcuma Species and the Isolation of Compounds from Curcuma aromatica Rhizome
Source: Biomolecules. 2020 May 21;10(5):799. doi: 10.3390/biom10050799 (PMC7277146; doi:10.3390/biom10050799)

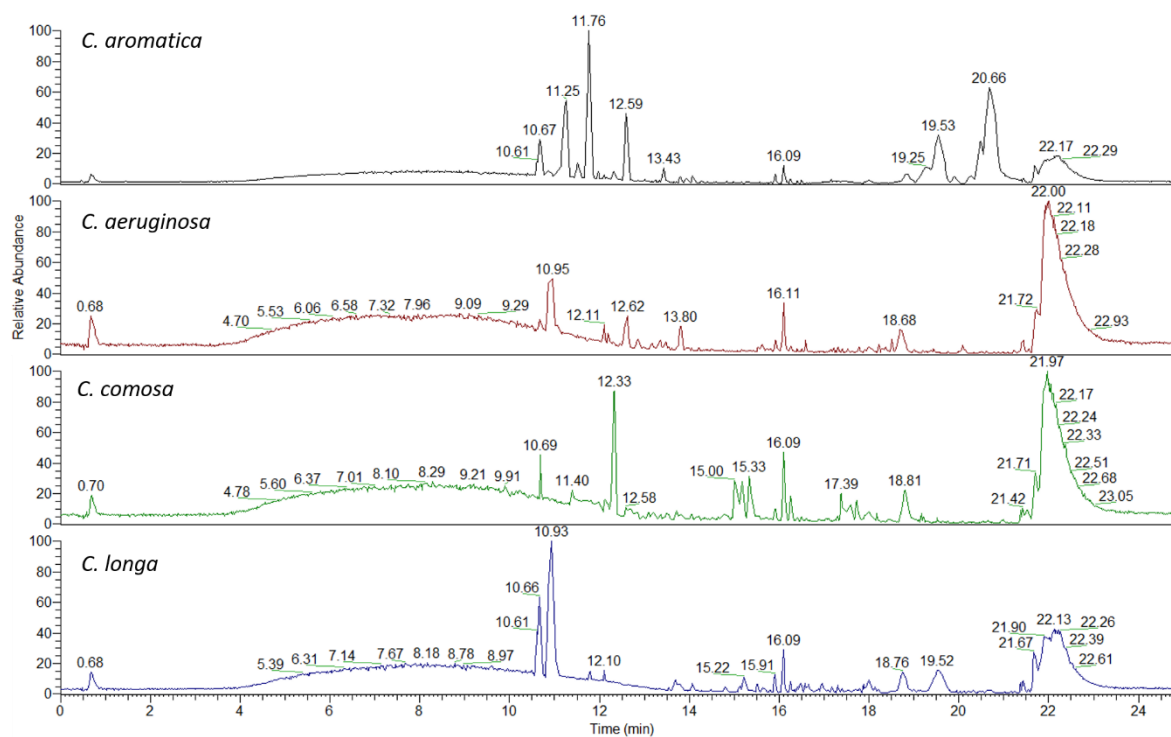

**Figure S1.** UPLC-HRMS chromatograms of four *Curcuma* extracts.

Supplement: Supplementary file 1 [file biomolecules-10-00799-s001.pdf]
